# Carole Hooven, Review of *T: The Story of Testosterone, the Hormone That Dominates and Divides Us*

**DOI:** 10.1093/emph/eoab043

**Published:** 2021-12-11

**Authors:** James R Roney

In *T*, Carole Hooven presents evidence for the importance of testosterone (hereafter ‘T’) in explaining human sex differences in a number of domains, as well as the importance of this hormone for regulating changes in morphology, psychology and behavior within-individuals over time. As an overall assessment, I thought the book was very well-written and presented a clear, accessible and accurate review of major themes in the human testosterone literature. The book introduced little in the way of original arguments or perspectives on this literature, however, and so may be of limited interest to readers who already have expertise in human behavioral endocrinology. For lay audiences, or for scientists who are seeking an initial introduction to the evidence for the role of T in human behavior, I think the book will be thought-provoking and informative, and I highly recommend it.

It is clear throughout the book that Hooven is arguing against other scholars who have argued that T has little relevance to explaining human behavioral or psychological sex differences. A main objective of the book is thus to correct misrepresentations of the scientific literature from these other sources, and more generally to marshal the overall evidence for the important causal effects of T. I think Hooven largely succeeds in these objectives. In organizing the evidence supporting the importance of T, though, Hooven at times glosses over subtleties and complications regarding the role of T specifically in humans, which might be considered a limitation of the book. In what follows, I attempt to give a sense of the type of case that Hooven makes to support the importance of T to human behavior, but then point out subtleties in the human research that make for a more complicated but also potentially more complete account of the role of this hormone.

## WEIGHT OF THE EVIDENCE

In the opening chapters, Hooven reviews some of the most vivid and persuasive examples that argue for effects of T on the development and sexual differentiation of the bodies of humans and other species. These include early animal experiments involving removal and reimplantation of testes, the ‘Castrati’ (boys castrated to prevent voice deepening to allow them to sing as sopranos), imperial Chinese eunuchs and complete androgen insensitivity syndrome (CAIS). In CAIS, T is unable to act through the androgen receptor: individuals with XY chromosomes who have the condition develop a predominantly female-typical phenotype, providing strong evidence for the role of T in producing male-typical sexual differentiation in humans. These examples provide overwhelming and memorable evidence for causal effects of T, and it was likely a good strategic decision to begin with them as a type of opening argument. Doing so sets up a rhetorical question: Is it likely that the effects of T are restricted only to the body outside of the brain and that T does not also affect brain development*?*

The idea that hormones affect physical development in humans but have minimal effects on the brain and behavior—which are instead the products of ‘socialization’—operates as a type of null hypothesis against which Hooven argues throughout *T*. She reviews evidence in nonhuman species showing that hormone manipulations can produce sex-atypical behaviors in domains ranging from juvenile play styles to adult sexuality, all under conditions in which rearing environments are experimentally controlled. Although analogous controlled experiments cannot be conducted with human participants, various lines of evidence support similar hormonal influences. Hooven reviews examples such as 5-alpha reductase deficiency and congenital adrenal hyperplasia in which early androgen exposure is atypical for the assigned gender, with phenotypic outcomes consistent with causal effects of T (other examples not directly discussed in the book are equally compelling, such as gender reassignments in infancy due to cloacal exstrophy or surgical accidents; see [1]). Skeptics of the importance of T can argue that subtle socialization effects associated with caregivers’ knowledge of atypical developmental conditions cannot be definitively ruled out in these cases, though as Hooven notes, there is no direct evidence for this position. The parallels between the human and nonhuman data, combined with theorized functions of hormones as signals that often coordinate morphological changes with behavioral strategies, together create a weight-of-the-evidence argument that strongly supports the importance of T effects in the human brain, as well as in the rest of the body.

Looming large in many of these arguments is the important distinction between organizational and activational effects of hormones. Organizational effects are roughly developmental effects that are largely irreversible, such as prenatal hormonal influences on genital development. Activational effects are more switch-like and reversible, such as antlers in red deer that grow when T is high in the breeding season, but atrophy and fall off when T is low. An important consideration when evaluating sex differences is that many phenotypic outcomes are affected by *both* organizational and activational effects. In some songbird species in which only males sing, for instance, T is often necessary to trigger adult singing behavior (an activational effect), but early life hormone exposure (derived from male testes) is also necessary for the development of the neural machinery that controls song (an organizational effect) [2]. Thus, administration of T to females in adulthood only is insufficient to trigger song since such females would not have been exposed to the necessary organizational hormone effects. Similar conjunctions of organizational and activational hormone effects appear necessary to explain outcomes such as sexual attraction to males versus females in many species.

The combined organizational and activational effects of hormones may create spaces of evidentiary ambiguity that lead to the disagreements about the importance of T that are addressed in *T*. Imagine that only activational effects existed. In that case, debates about the importance of T might have easy empirical resolutions: if T were proposed to cause a specific behavioral or psychological sex difference, one could simply block or administer T to test whether the sex difference could be reversed. If T has both organizational and activational effects, though, then such tests are largely precluded by the difficulty of manipulating (or even measuring) early hormone exposures in humans.

Hooven writes that the T skeptics whom she argues against often draw unwarranted inferences about the influence of T by pointing to small within-sex correlations between T and various outcomes when both are measured in adulthood. For strength and athletic performance, for instance, she argues that others have incorrectly inferred from very small within-sex correlations between T and athletic performance that between-sex differences are not caused by sex differences in T exposure. Part of this is an argument about activational effects: Since adult men and women show almost no overlap in their distributions of T production, higher T could explain greater strength in men versus women even if it explains little variation within each sex. (Note, however, that the lack of overlap makes this difficult to test statistically: between-sex T differences are confounded with any other differences between the sexes.) But I think the real issue here concerns the complications introduced by organizational effects, especially those that occur at puberty. Hooven quite nicely summarizes a suite of effects that T has during pubertal development that should have lasting effects on athletic performance, such as increasing bone size and density, enhancing body height, increasing skeletal muscle mass, affecting hip width and so on. Since men experience much greater T production during puberty, these effects could persist even if T were manipulated in adulthood, thus precluding simple tests of whether T is causing sex differences in strength and athletic performance. In lieu of dispositive empirical tests, then, we are left again with weight-of-the-evidence arguments that T exposure across the entire lifespan is highly likely to explain substantial fractions of the measurable average sex differences in muscle mass, strength and athleticism.

Similar weight-of-the-evidence arguments are made to argue for causal effects of T on sex differences in aggression and sexuality. I think these arguments are generally persuasive, though again, within-sex correlations between T and the variables of interest tend to be small and inconsistent when measured during adulthood. Those within-sex correlations raise some questions. Why, for instance, is the within-sex correlation between T and strength/athletic performance so low given the importance that Hooven attributes to T for this and many other outcomes? This is where I think things start to get more complicated.

## EVERYTHING IS COMPLICATED

Throughout the book, Hooven presents T as a signal that promotes a number of specific outcomes, especially in males: greater muscle mass, aggression, sexual desire and so on. In some sense, her argument for the importance of T in humans seems to rely on its associations with these specific phenotypic outcomes. Hooven touches on why one signal might coordinate this specific group of outcomes when she discusses mate competition in seasonally breeding species like red deer: T rises in the breeding season when fertile females are present, and then essentially activates a suite of coordinated behavioral (aggression and sexual motivation) and morphological (antlers and muscle mass) outcomes that together all promote competition for mating opportunities. In the nonbreeding season when females cannot conceive, T drops to avoid the costs of these outcomes (ranging from energy use to risk of injury). Thus, we can see T in an abstract functional sense as a signal that shifts investment of behavioral and energetic effort between mate competition and alternative adaptive priorities.

Elsewhere, I have argued that we can build functional theories about hormones by constructing what I call ‘theoretical frameworks’ for them [3]. Theoretical frameworks are essentially maps that list input conditions to a hormone on one side of the map (e.g. cues of season like photoperiod in the red deer example) and list coordinated output effects of the hormone on the other side (e.g. antler growth, increased aggressiveness in the same example). Importantly, however, specific inputs and outputs can change across species in response to changes in selection pressures. In species that are not seasonally breeding, for instance, cues of season may not act as inputs to changes in T production. Likewise, in species for which direct combat between males is less significant to mate competition, output effects of T may diminish for combat-related traits. Viewed through the lens of theoretical frameworks that evolve gradually over time, hormones like T can be expected to retain some input–output relationships seen in other species, but to also exhibit species-specific changes in these patterns.

The evolution of pair bonding and paternal provisioning of offspring may have altered some of the outputs of T in humans relative to many other mammals. Hooven reviews evidence that is consistent with T functioning to allocate investment in mate competition versus other priorities in human males, such as the robust finding that T drops in men after they form pair bonds or become fathers. This drop in T may reduce motivation to seek other mates when paternal provisioning is important, though the evidence for this is more indirect in humans than in other paternally investing species. But a question arises about effects of T on muscle mass and strength: is it functional to have these outcomes decline after men become fathers?

Alvarado et al. [4] proposed the ‘Paternal Provisioning Hypothesis’, which postulates that the importance of muscle mass and strength for male-dominated paternal provisioning activities (such as large game hunting) led to a weakening of the relationship between T and strength in humans. They demonstrated in a rural community that men increased their agricultural workloads after having kids, and that this in turn increased their strength and muscle mass despite a paternity-associated decline in T. Furthermore, controlling for age, workloads and paternal status, regression models showed no significant associations of T with strength or muscle mass. A review of the overall literature likewise showed that T accounts for tiny fractions of the between-person variance in muscle mass in humans, as opposed to other primates for which it explains greater fractions of this variability [4]. The Paternal Provisioning Hypothesis provides a potential answer to the question of why within-sex relationships between T and strength/athleticism may be so weak: a specific output effect of T decreased in strength relative to other primates due to selection pressures associated with paternal effort in humans.

Similar complications arise regarding the relationship between T and sexual desire. In many nonhuman species, sexual desire in males drops to zero when females cannot conceive, as during the nonbreeding season, or even during nonfecund regions of female estrous cycles. In humans, however, concealed ovulatory timing combined with pair bonding may have selected for a male sexual psychology that produces desire for sex with partners at fairly regular intervals in order to catch concealed insemination opportunities whenever they happen to arise. Because T declines when men enter relationships, however, the mapping of T to sexual desire may also have declined in humans, such that only minimal threshold amounts of T are necessary to fully maintain sexual desire [see 5]. As such, as with strength and muscle mass, the relationship between T and sexual motivation may be more nuanced in humans than it is in other species.

None of this is to suggest that Hooven inaccurately characterizes any of these patterns, but only that *T* does not delve into these nuances. When discussing the relationship between T and sexual motivation in men, she writes: ‘…we know that large increases in men’s T levels, going from extremely low to normal, will increase sex drive, sexual arousal, and sexual function. And the reverse is true’ (p. 197). This is accurate and consistent with the minimal threshold effects referred to above, but it omits evidence that increasing T from average to high concentrations appears to have no clear effects on these same variables [reviewed in 5]. Such omissions may occur because Hooven is in general arguing against skeptics who state or imply that T has little to no importance at all for human behavior and psychology, but subtleties in the effects of T specifically in humans are glossed over in the process of arguing against the skeptics’ positions.

This brings us back around to the larger debates that animate this book. When one considers evidence that within-sex correlations between T and variables like sexual desire, strength and aggressiveness are all quite small, one can understand why some scholars have argued that the importance of T for human behavior has been exaggerated. Hooven can counter that when organizational effects of hormones are considered in conjunction with activational effects, the weight of the evidence supports an important role for this hormone in explaining many human sex differences, as well as within-individual changes over time. I think those arguments are correct, but they are unlikely to persuade the T skeptics given the difficulty of conducting research on organizational hormone effects in humans. Finally, what I have tried to contribute here is that the reasons those within-sex correlations are so small is itself part of a broader story regarding evolutionary changes in the functional roles of T in humans. Those changes do not make the roles of T any less important or interesting, and in fact, are related to some of the key evolutionary changes from other primates that make us uniquely human.
